# Kimura's Disease: A Rare Cause of Unilateral Tonsillar Enlargement

**DOI:** 10.1155/2021/8815317

**Published:** 2021-01-07

**Authors:** Prakash Khanal, Agya Shrestha

**Affiliations:** Nepal Police Hospital, Maharajgunj, Kathmandu, Nepal

## Abstract

**Introduction:**

Kimura's disease is a rare inflammatory disorder of unknown cause, commonly seen in young Asian males. *Case Report*. A 61-year-old male patient presented with a history of right tonsillar mass and cervical lymphadenopathy. The patient underwent hematological investigation and imaging followed by resection of tonsillar mass. Based on histopathological and subsequent immunohistochemistry reports, the case was diagnosed as Kimura's disease of the tonsil. *Discussion*. Kimura's disease commonly presents as painless subcutaneous masses in the head and neck region or cervical lymphadenopathy. Kimura's disease presenting as a tonsillar mass is a very rare condition. Patients usually have peripheral eosinophilia and elevated levels of serum IgE. The diagnosis is based on the clinical and histopathologic findings in a biopsy of the mass and/or lymph node along with elevated peripheral eosinophil and serum IgE level.

**Conclusion:**

The clinical presentation of Kimura's disease is highly variable. Kimura's disease should be considered as a differential diagnosis in patients presenting with a tonsillar mass. A high index of suspicion along with histopathological examination helps in the early diagnosis and management. Surgical excision is the treatment of choice.

## 1. Introduction

Kimura's disease is a chronic inflammatory disease of unknown etiology [1]. This disease typically occurs in young Asian male patients in their second to fourth decades of life, although rare cases in other races and ethnicities have also been seen [2]. The exact prevalence of Kimura's disease is not known. Around 200 cases have been reported worldwide till date [3]. Most of these cases are reported in East and Southeast Asia, with a small number of cases reported in Europe and the Middle East [4, 5].

Kimura's disease commonly presents as subcutaneous masses in the head and neck region or cervical lymphadenopathy [6]. Patients usually have peripheral eosinophilia and elevated levels of serum immunoglobulin E (IgE) [7]. Kimura's disease of the tonsil is a very rare condition. We report a rare case of Kimura's disease presenting as a unilateral tonsillar enlargement in an elderly man in Nepal. The clinical presentation, diagnosis, and treatment options for this rare disease are discussed in this article.

## 2. Case Report

A 61-year-old man presented to the ENT outpatient department with a history of a right tonsillar mass for 1 month. There was no history of throat pain, fever, or weight loss. The other medical and family histories were not significant.

**Figure 1:**
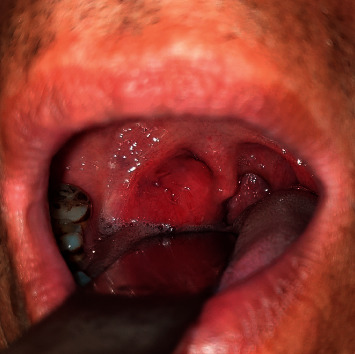
Picture showing the right oropharyngeal mass.

**Figure 2:**
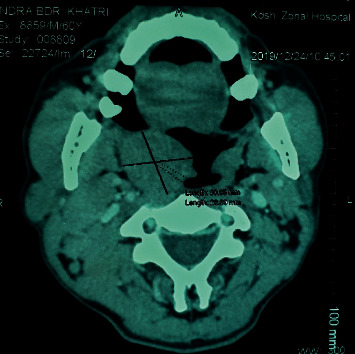
CT scan showing well-defined minimally enhancing isodense soft tissue mass in the oropharynx.

On physical examination, there was a 3 × 2 cm, nontender, mobile mass arising from the right lateral wall of the oropharynx (Figure 1). Few palpable lymph nodes were present at right levels III and IV. Hematological investigation revealed mild eosinophilia (9%). Contrast-enhanced computerized tomography (CT) scan of the neck revealed well-defined minimally enhancing isodense soft tissue mass (31 × 27 mm) in the oropharynx on the right side (Figure 2). Fine needle aspiration cytology (FNAC) from cervical lymph nodes revealed reactive lymphadenitis. The patient underwent R0 resection of the oropharyngeal mass. Postoperative period was uneventful.

**Figure 3:**
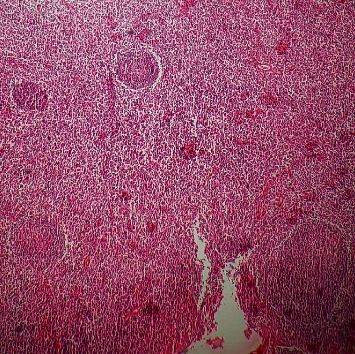
Lymphoid follicles within the tissue (H&E stain, ×100).

**Figure 4:**
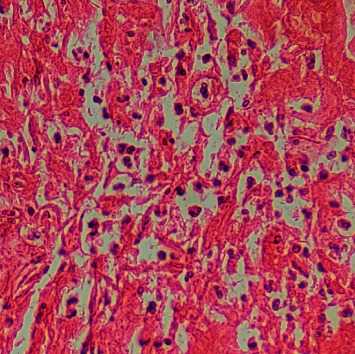
Eosinophil infiltration within the tissue (H&E stain, ×400).

**Figure 5:**
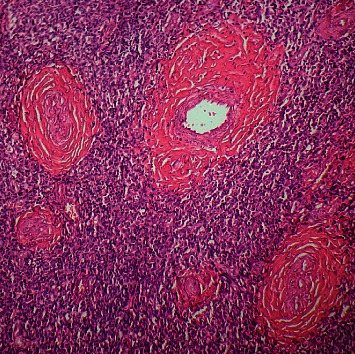
Tissue showing hyalinized blood vessels (H&E stain, ×100).

Histopathological examination of the specimen showed multiple lymphoid follicles with germinal centers and eosinophilic infiltration (Figures 3 and 4). The vascular component consisted of hyalinized blood vessels lined by flat endothelial cells (Figure 5). The findings were suggestive of Kimura's disease. The patient was advised for immunohistochemistry (IHC) to confirm the diagnosis. On IHC, the cells were positive for CD3, CD5, CD20, CD23, and CD45. Germinal center cells were positive for CD10, and BCL2 was positive in mantle zone cells and paracortical areas. A serum IgE level was measured, which was elevated at 276 kU/L (normal: 0–75 kU/L), which further supported the diagnosis of Kimura's disease. Final diagnosis of Kimura's disease was made based on these findings.

## 3. Discussion

Kimura's disease is a chronic inflammatory disorder of unknown etiology. This disease was first described by Kim and Szeto as “eosinophilic hyperplastic lymphogranuloma” and later was named Kimura's disease by Kimura et al. [8]. It is more prevalent in the Asian population; few cases have been reported in the Western world. The disease mostly occurs in the second and third decades of life with male predominance [2]. Kimura's disease usually presents as painless subcutaneous masses in the head and neck region or cervical lymphadenopathy. Extracutaneous sites of involvement include the parotid, orbit, oral cavity, and paranasal sinuses [9–11]. Kimura's disease arising from tonsil is a very rare condition. On reviewing the literature studies, we found a single case report of Kimura's disease arising from palatine tonsil published by Matsumoto et al. [12].

Most of the patients with Kimura's disease have peripheral blood eosinophilia and elevated serum IgE levels [13]. Study by Horikoshi et al. [14] reported a mean eosinophilia count of 35.2% with increased IgE levels in all Kimura's disease cases. Our patient had an eosinophil count of 9%. Systemic symptoms are usually absent. Renal involvement may present itself as glomerulonephritis and nephritic syndrome.

The pathogenesis of Kimura's disease is unknown. Trauma, infection, type I hypersensitivity reaction, and autoimmune process have been postulated as possible causes. Infection or toxin may trigger an autoimmune reaction or lead to a type I hypersensitivity reaction [9].

The diagnosis of Kimura's disease is based on the clinical findings and histopathologic analysis of the mass and/or lymph node. Computerized tomography (CT) and magnetic resonance imaging (MRI) may be useful to determine the size and depth of lesions and the involvement of surrounding tissues and assist in surgical planning [10–14].

Kimura's disease is one of the rare causes in the differential diagnosis of a tonsillar mass with lymphadenopathy. Other differential diagnoses include non-Hodgkin lymphoma, squamous cell carcinoma, Hodgkin's disease, leukemia, metastatic neoplasms, and other infectious and inflammatory causes of tonsillar enlargement. The clinical, radiological, and histological features help to differentiate them from Kimura's disease.

Kimura's disease has a benign course. The preferred treatment for localized Kimura's disease is surgical excision. Other treatment approaches include surgical excision with postoperative low-dose radiation therapy, radiation therapy alone, and medical therapy [15, 16]. Systemic steroids have good effects on disease progression; however, withdrawal can often result in relapse. Steroid-resistant lesions can be treated with radiation. Recurrence is common after any type of treatment. Our patient was managed with surgical excision of the mass. He was asymptomatic for one year after the surgery without recurrence. The overall prognosis of Kimura's disease is good. No cases of malignant transformation have been reported in the literature.

## 4. Conclusion

Kimura's disease of the tonsil is a very rare disease. To the best of our knowledge, this is the second case reported in the literature so far. Diagnosis can be difficult due to its rare occurrence. Kimura's disease should be considered as a differential diagnosis in patients presenting with tonsillar mass and investigated accordingly. Surgical excision is the effective treatment in localized Kimura's disease.

## Data Availability

The data supporting the results are not available for readers to review as they contain confidential patient health information.

## Supplementary Materials

Supplementary Materials The supplementary file includes the CARE checklist.
